# A Review of Circulating Tumor DNA in Hepatobiliary Malignancies

**DOI:** 10.3389/fonc.2018.00212

**Published:** 2018-06-11

**Authors:** Kabir Mody, Sean P. Cleary

**Affiliations:** ^1^Division of Hematology/Oncology, Mayo Clinic Cancer Center, Mayo Clinic, Jacksonville, FL, United States; ^2^Division of Hepatobiliary/Pancreas Surgery, Mayo Clinic Cancer Center, Mayo Clinic, Rochester, MN, United States

**Keywords:** cholangiocarcinoma, hepatocellular carcinoma, circulating tumor DNA, liver neoplasms/blood, liver neoplasms/genetics

## Abstract

Circulating tumor DNA (ctDNA) is released into circulation (blood) specifically from tumor cells undergoing metabolic secretion, apoptosis, or necrosis, carries tumor-specific genetic or epigenetic alterations. Technologies enabling clinical evaluation of ctDNA continue to advance rapidly and allow for the assessment of patient-specific tumoral genetic and epigenetic alterations. This holds great potential for earlier detection of disease, serial monitoring of tumor heterogeneity, identification of therapeutic targets, and evaluation of treatment response and mechanisms of resistance. Hepatobiliary malignancies are often diagnosed late, recur commonly, yield limited available tumor on biopsy, and harbor several genomic alterations with potential therapeutic impacts. Patients suffering from or at risk for these diseases thus stand to benefit immensely from this technology. Herein, we review the limited literature pertaining to the potential for ctDNA technologies in such patients. Patients with these cancers stand to benefit greatly from the application of ctDNA technologies, and concerted efforts at further investigation of such are ongoing and greatly needed.

## Introduction

**Figure 1:**
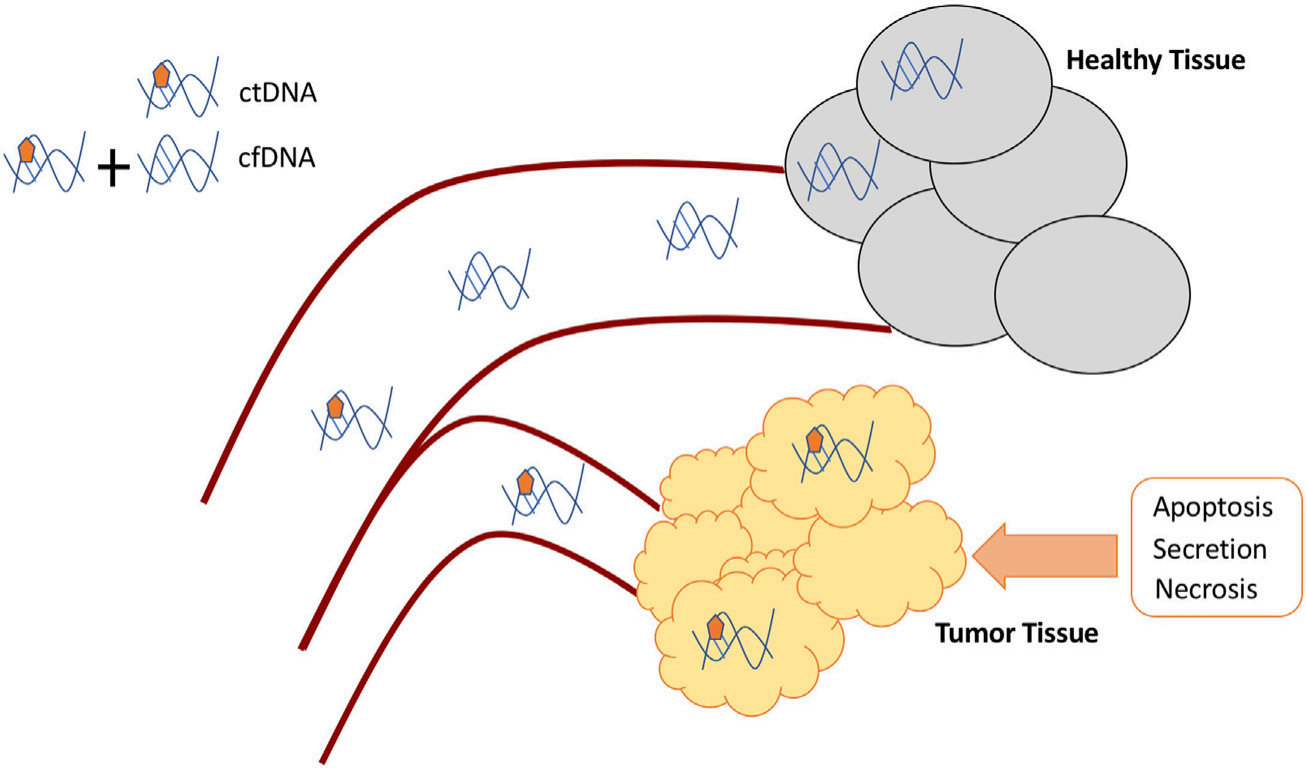
Circulating DNA generation and differences.

Circulating genetic material is made up of exosomes, tumor-educated platelets, circulating tumor cells, microRNA, and cell-free DNA (cfDNA) (1, 2). The content of cfDNA is predominately short, double-stranded fragments of nuclear and mitochondrial DNA. While healthy individuals have cfDNA detectable in their serum that is released from normal cellular processes, the cfDNA in cancer patients is composed of DNA fragments released from normal and cancer cells. Circulating tumor DNA (ctDNA) is a component of cfDNA found in cancer patients composed of DNA released into circulation specifically from tumor cells that undergo metabolic secretion, apoptosis, or necrosis (Figure 1). Serum samples generally yield more cfDNA, but the additional material above and beyond ctDNA is derived from, for example, leukocyte lysis during clotting, which thus dilutes the ctDNA content. There are various techniques available to extract ctDNA from the plasma of cancer patients, and these methods vary in their ability to purify fragments of different sizes, thus changing the total quantity of cfDNA isolated and the fraction of ctDNA captured (3). Discriminating ctDNA from normal cfDNA is aided by the fact that tumor DNA is defined by the presence of mutations. These mutations are present only in the genomes of cancer cells or precancerous cells and are not present in the DNA of normal cells. This affords ctDNA significant biologic specificity as a biomarker (4, 5). The ability to detect and characterize ctDNA enables a wide array of practical clinical applications that are not possible with routine sequencing of tumor tissue or with other circulating biomarkers (4).

Circulating tumor DNA carries tumor-specific genetic or epigenetic alterations, such as point mutations, copy number variations, chromosomal rearrangements, and DNA methylation patterns. PCR-based (digital PCR) and next-generation sequencing (NGS)-based methods are two dominant approaches in this field for analysis of ctDNA (5). Digital PCR approaches are highly sensitive but can only examine a single or a few mutations of interest at any one time. Sequencing-based approaches have the ability to look at a number of genes at a whole-genome or whole-exome level; however, these techniques are currently limited due to detection rates that approach error rates of PCR and sequencing technology. Capture-based NGS has the ability to enrich genomic regions of interest by hybridizing target genes/regions to antisense oligonucleotides before sequencing; this approach allows for agnostic analysis of large portions of the genome and can identify multiple mutations with increased sensitivity (6).

The evaluation of ctDNA enables assessment of patient specific tumoral genetic and epigenetic alterations and offers a unique opportunity for serial monitoring of tumor genomes in a non-invasive, convenient, and accurate manner. Potential applications of ctDNA testing in patients with cancer include (a) early detection of disease, (b) monitoring of tumor heterogeneity, (c) identification of therapeutic targets, (d) real-time evaluation of treatment response and tumor relapse, and (e) real-time assessment of evolution of drug resistance (4). Along with significant advancements of sequencing technology in recent years, an equal effort and investment are underway to optimize ctDNA use for routine clinical practice.

Hepatobiliary (HPB) malignancies including hepatocellular carcinoma (HCC) and cholangiocarcinoma (CCA) stand to gain immensely from the use of ctDNA given that (a) diagnosis currently is more often made at advanced stages of disease, (b) recurrences are common despite pursuit of potentially curable treatments such as surgery, (c) biopsies are not always obtained or often yield suboptimal quantities of tumor cells and thus insufficient tumor DNA for tissue-based genomic profiling, and (d) multiple genomic alterations which are targetable with therapeutics currently in the clinic demonstrating significant efficacy are known to occur in disease such as CCA.

## Hepatocellular Carcinoma

Hepatocellular carcinoma is a lethal liver malignancy with an exceptionally high incidence in Asia and Africa. The number of new cases in many countries is rapidly increasing, making HCC a worldwide health problem (7).

The diagnosis of HCC can often be made using non-invasive imaging such as ultrasonography, computed tomography (CT), and magnetic resonance tomography (MRI), aided by the use of LiRADS criteria, along with the measurement of alpha-fetoprotein (AFP) level, a predictive biomarker for HCC (8). Given the ability to use non-invasive techniques to make a diagnosis, invasive biopsy is less commonly pursued and considered to make a diagnosis of HCC only when imaging tests are less confident in a particular case. As a result, pre-treatment tissue sampling is rarely available for genomic profile analysis. However, imaging tests can only determine HCC with confidence when nodules grow to over 1 cm in size. In addition, the use of AFP to aid in a confident diagnosis is not always possible given that not all HCC can produce elevated levels of AFP (9).

Early-stage HCC are currently difficult to diagnose and characterize, but can be effectively treated by surgical resection with a 5-year survival rate of 90% (10). Other than surgical resection, several options exist for definitive management of disease including liver transplantation, transarterial chemoembolization, radiofrequency or microwave ablation, or radioembolization. Unfortunately, however, a considerable proportion of patients are still diagnosed with advanced disease for which treatment options have been limited and prognosis remains poor.

### Early Detection

A large part of the potential of ctDNA use in cancer, is the possibility to use it for earlier detection of disease thus enabling institution of more effective, potentially curative treatment approaches. In the case of HCC, a few studies have evaluated ctDNA use for this purpose by evaluating the ability to detect ctDNA-specific genomic alterations linked with HCC (Table 1). Ser249 of *TP53* has been the most reported mutation hotspot in HCC patients, and mutation of this site leads to a defect in TP53-specific DNA-binding ability (11–13). Huang et al. demonstrated the ability to detect, in ctDNA, the presence of this mutation in patients residing in the Qidong area of China exposed to aflatoxin and with high prevalence of hepatitis B virus carriers. The mutation was found in 40% of HCC cases, 20% of cirrhotics, and 7% of healthy controls, with an adjusted odds ratio of 22.1 for HCC cases compared with controls. They suggested that the detection of this mutation in ctDNA testing was potentially a method for early diagnosis in this population (14). The presence of the same mutation was evaluated in ctDNA in a population of patients with similar exposure to aflatoxin and hepatitis B in Gambia, Africa. This group, in particular, compared the tissue and ctDNA detection rates of this particular mutation and noted a concordance between tumor tissue and matched plasma of 88.5% (15). A group in Egypt also examined the presence of the same TP53 mutation, in addition to mutations in CTNNB1, in cfDNA of patients with HCC or chronic liver disease. Circulating DNA concentrations were significantly higher in HCC patients compared with HBV and HCV carriers without cancer, and to seronegative individuals. However, their results regarding detection of Ser249 TP53 mutations did not parallel those from prior studies (16). Interestingly, this Ser249 mutation has also been detected in noncancerous hepatic tissues of HCC, in the plasma DNA of a minority of healthy individuals, and in patients with relatively more severe cirrhosis (17, 18). Importantly, these results highlight the potential of ctDNA as a part of early detection strategies for particular populations at higher risk for HCC, though clearly much work is necessary to identify sensitive genomic targets in particular high-risk populations, and to validate these alterations as highly sensitive targets for enabling early diagnosis.

**Table 1:** Circulating DNA biomarkers explored in hepatocellular carcinoma.

Early detection	Diagnosis/prognosis
TP53 Ser249	Cell-free DNA levels
CTNNB1	GSTP1
	hTERT
	TP53

### Diagnosis and Prognosis

In addition to its potential in the setting of early diagnosis of disease, evaluation of ctDNA has potential as a tool to assist with the diagnosis and prognostication of HCC at other stages of disease too, including diagnosis of disease in particular higher risk populations. In addition, given the risk of HCC recurrence after potentially curative treatment strategies, such as surgery, there may be value to the use of ctDNA in post-treatment surveillance. The value of circulating DNA evaluation in regard to prognosis has also been evaluated. Both circulating DNA level and the presence of specific gene alterations have been shown to be potential prognostic markers, indicating higher risks of disease recurrence and shorter survival (Table 1).

First, in the realm of using circulating DNA as a diagnostic tool, one study evaluated a cohort of 96 patients with HCV-related HCC and in 100 HCV carriers without known HCC and validated the finding that serum cfDNA levels were significantly higher in HCC patients than in HCV carriers (*P* < 0.0001). To investigate the value of circulating DNA when combined with other blood-based biomarkers, another study evaluated the power of combined detection of circulating cfDNA, AFP, and α l-fucosidase (AFU) for diagnosis of HCC in serum samples from 39 HCC patients and 45 normal controls. cfDNA levels in HCC patients were significantly higher than that in normal controls (*P* < 0.05). Quantitative analysis of cfDNA was found to be sensitive and feasible, and the combined detection of cfDNA with AFP or AFU or both was found to improve the diagnostic sensitivity for HCC (19). A meta-analysis evaluating published results regarding qualitative and quantitative analyses of circulating cfDNA in HCC and the use of cfDNA values for HCC diagnosis investigated three subgroups: qualitative analysis of abnormal concentrations of cfDNA, qualitative analysis of single-gene methylation alterations, and multiple analyses combined with AFP. A total of 2,424 subjects included 1,280 HCC patients in 22 studies were included. The pooled sensitivity and specificity of quantitative analysis were 74 and 85%, respectively. For qualitative analysis, the sensitivity and specificity were 53.8 and 94.4%, respectively. After combining with AFP assay, capabilities improved, with the values being 81.8 and 96%, respectively (20).

As a diagnostic tool Iizuka et al. evaluated the use of a real-time PCR assay for levels of the glutathione *S*-transferase pi (GSTP1) gene in cfDNA in the blood of 52 patients with HCC associated with HCV, 30 HCV carriers without known HCC, and 16 HCV-negative non-cancer patients. cfDNA levels were significantly higher in HCC patients than in HCV carriers or the control subjects with a sensitivity of 69.2% and a specificity of 93.3% in discriminating HCC and HCV carriers (21). Another study sought to evaluate the use of cfDNA, focused on a particular gene, hTERT, as a diagnostic and prognostic tool in HCC. In 142 plasma samples obtained from 66 patients with HCC, 35 with cirrhosis, and 41 with advanced HCV-related chronic hepatitis, cfDNA was documented in the plasma of 22% of chronic hepatitis patients, 57% of those with cirrhosis, and 61% of HCC patients. Patients with multinodular HCC showed significantly higher levels of cfDNA (*P* = 0.05), and survival was significantly longer in patients with cfDNA below than in those above the cutoff value (37 versus 24 months, *P* = 0.03) (22).

In regard to the use of circulating DNA in the post-operative setting, another study evaluated cfDNA levels in 87 patients who had undergone curative-intent hepatectomy for HCC. They found that those with a high cfDNA level post-operatively had a significantly shorter overall survival (OS) time compared with those in whom the cfDNA level was not high. cfDNA level was determined to be an independent prognostic factor for OS and cancer recurrence in distant organs (23). Ono and colleagues enrolled 46 patients with HCC who underwent hepatectomy or liver transplantation and evaluated the cumulative incidence of recurrence and extrahepatic metastasis in the ctDNA-positive group, noting that it was statistically significantly worse than in the ctDNA-negative group (*P* = 0.0102 and 0.0386, respectively) (24). Another study evaluated a gene-specific approach, seeking to specifically detect p53 mutations in the cfDNA of transplanted HCC patients and to determine the utility of this method in the diagnosis of HCC tumor recurrence. In a group of 24 liver-transplanted HCC patients, compared with a group of healthy controls, it was indeed (a) possible to detect mutated p53 genes in cfDNA and (b) this was noted to be useful as a biomarker of tumor recurrence during the clinical evolution of transplanted patients (25). In yet another study, Ren et al. sought to quantify the circulating DNA in pre-operative plasma from 79 patients with HCC before operation, 20 patients with liver cirrhosis, and 20 healthy volunteers, and assess for an association between circulating DNA level and prognosis of HCC patients. Circulating DNA level was closely associated with tumor size (*P* = 0.008) and TNM stage (*P* = 0.040) and was negatively associated with the 3-year DFS (*P* = 0.017) and OS (*P* = 0.001) (26).

### Treatment

Perhaps one of the most exciting and explored areas of potential for ctDNA across cancer types has been as a more non-invasive, comprehensive tool to enable precision medicine as a therapeutic reality for some patients. As regards the use of circulating DNA for the purposes of treatment, work has been ongoing to optimize cfDNA/ctDNA’s capabilities to provide comprehensive genomic profiling of potential therapeutic targets and also to monitor disease response on treatment. To enable its use in clinic on a routine basis, it is necessary to prove high concordance with the gold standard, tissue-based profiling, for one. Little has been reported in the literature with regard to tissue and circulating DNA mutation analysis concordance, unfortunately. In one study, from data in 105 patients with GI malignancies, some with HCC, overall concordance rates of 96, 94, 95, and 91%, respectively, were found between ctDNA and tissue biopsy in the four most common alterations (KRAS amplification, MYC amplification, KRAS G12V, and EGFR amplification) (27). One small study performed whole-exome sequencing and targeted deep sequencing (TDS) in 32 multiregional tumor samples from five patients. Matched cfDNA was sequenced accordingly. Although the genome profiling efficiency of cfDNA increased with sequencing depth, an average of 47.2% total mutations were identified using TDS, suggesting that tissue samples outperformed it. Optimistically, 38.6% of patients carried mutations that were considered potential therapeutic targets (28). Focusing on 574 cancer genes known to harbor actionable mutations, another small study in 3 patients identified the mutation repertoire of HCC tissues and monitored the corresponding ctDNA features in blood samples to evaluate its clinical significance. Analysis revealed that ctDNA could overcome tumor heterogeneity and also provided information regarding tumor burden and prognosis. Analysis on a fourth HCC case with multiple lesion samples and sequential plasma samples identified 160 subclonal SNVs in tumor tissues and matched peritumor tissues with PBMC as control. 97% of this patient’s tissue mutations could be also detected in plasma ctDNA. Many mutations also showed circulating levels correlating to cancer progression (29).

In terms of evaluating the landscape of genomic alterations in HCC, through the eyes of ctDNA, again there are limited reported data. A notable recent study in 213 patients with advanced gastrointestinal cancers sought to assess the utility of ctDNA detection across a panel of 68 genes with a commercially available assay, with HCC patients representing 15% of the study’s population. The majority of patients (58%) had >1 characterized alteration (excluding variants of unknown significance), with a median number of characterized alterations being 1 (range, 0–13). The number of detected alterations per patient varied between different cancer types: in HCC, 74% of patients had >1 characterized alteration, versus 24% of appendiceal adenocarcinoma patients. Of the 123 patients with characterized alterations, >99% had one or more alterations potentially actionable by experimental or approved drugs. These observations from this large study suggest that many patients with gastrointestinal tumors, including difficult-to-biopsy malignancies like hepatocellular cancers, frequently have discernible and pharmacologically treatable ctDNA alterations (27).

Overall, the existing literature is still quite limited but, with this caveat, thus far demonstrates that the use of ctDNA for genomic profiling in HCC is feasible and may provide a tissue biopsy-free alternative in these difficult-to-biopsy patients. That being said, further study of the clinical validity and utility is needed.

## Cholangiocarcinoma

Cholangiocarcinomas are malignant tumors arising from cholangiocytes that form the epithelium of the biliary system (30). Tumors are traditionally classified by location as intrahepatic (iCC), perihilar (pCC), and extrahepatic (eCC) based on their presumed site of origin within the biliary ducts. While this anatomic classification seems simplistic, it is effective in differentiating biliary tumors in terms of epidemiology, etiology, clinical presentation, and treatment (30). As with HCC, early diagnosis is ideal given that surgical resection or liver transplantation, offers the patient the best chance at cure. However, the majority of patients diagnosed with this malignancy have advanced stage disease precluding surgical management.

While CCA is a rare malignancy accounting for approximately 3% of gastrointestinal cancers, its incidence has been rising steadily in the US (31–33). The disease is more prevalent in many countries of the Asian continent especially. Several risk factors for CCA have been described with most etiologies producing increased risk for cancer associated with long-standing inflammation (33). In Asia, long-standing biliary inflammation due to infection with biliary flukes *Opisthorchis viverrini* and *Clonorchis sinensis*, as well as chronic hepatolithiasis, are commonly associated with CCA. Chronic hepatitis C and B infection are also known to increase the risk for CCA. In Western countries, long-standing inflammation associated with primary sclerosing cholangitis (PSC), fatty liver disease, cholelithiasis, and smoking all are associated with increased risk (33).

### Diagnosis and Prognosis

The diagnosis of CCA can be challenging. Cross-sectional imaging using a combination of ultrasound, CT, and magnetic resonance imaging (MRI) is often important for lesion identification and localization. Blood-based biomarkers, most commonly Ca19-9, may also be helpful though they are elevated in just 60–65% of CCA patients. The utility of this marker is also limited by the large number of CCA patients with normal CA19-9, as well as elevations seen in a number of benign conditions such as PSC and biliary obstruction (34). Histologic confirmation of malignancy can be challenging, particularly in patients with PSC and biliary strictures. Brushings and biliary cytology can be occasionally obtained through endoscopic cholangiography, but its clinical yield can be low and insufficient especially for DNA extraction to enable genomic profiling. Pathologic interpretation of the cytology can be challenging particularly in the presence of inflammation. In addition, the desmoplastic nature of many CCA tumors also contributes to limitations of yield. For the above reasons, the potential is great for ctDNA as a means of diagnosis, in addition to prognosis.

The use of ctDNA in the diagnosis of CCA has particular interest due to the difficulty in diagnosing this malignancy in patients with inflammatory conditions and/or strictures. Obtaining sufficient cytologic material to confirm a cancer diagnosis is challenging, let alone acquiring enough additional material with which to perform genomic analyses. Andersen and Jakobsen utilized a multiplex digital PCR assay to screen for 31 mutations in *KRAS, NRAS, BRAF*, and *PIK3CA*. The accuracy of the assay was first confirmed in pooled normal serum and positive controls developed by site-directed mutagenesis (35). The authors then conducted the assay on serum of five CCA patients with known tumor mutations and 6 patients who were known to be wild type for the assayed mutations. The assay correctly identified the five known mutations while none of the six wild-type samples had mutations identified in cfDNA. While this multiplex mutation analysis appears to have good results for cfDNA, the applicability of this assay for CCA may be limited since the frequency of *KRAS, BRAF*, and *PIK3CA* are just 12, 4, and 6%, respectively, in The Cancer Genome Atlas (TCGA) analysis (36).

Investigations into the use of ctDNA in CC have been hampered by the rarity of the disease and the relatively incomplete understanding of the genetics of this cancer. The recent characterization of the CCA genome by several studies including that utilizing data from a commercially available tissue-based assay (37), in addition to TCGA analysis, has not only enhanced our understanding of the breadth of targetable somatic alterations in this cancer but also identified important target genes and subsets of tumors based on molecular profile (36 37). In fact, based on this work, a number of novel-targeted therapeutics have emerged and are in clinical trials for patients with CCA.

Genomic alterations in FGFR2 are found in up to 40% of CCAs. The most common form of alteration is a gene fusion products that join the 5′ exons containing the kinase domain to 3′ partner genes with fusions to *BICC1, AHCYL1, TACC3, MGEA5, KIAA1598, FRK, PPHLN1*, or *C10ORF118* (38). Goyal et al. analyzed ctDNA collected by serial sampling in patients enrolled in a Phase 1 study of BGJ398, a FGFR inhibitor (39). Among 32 patients screened, 9 (28%) had FGFR2 fusions detected and 4 patients were enrolled in the trial. Sequencing of the FGFR portion of the fusion genes were compared at enrollment and after progression in three cases. In all three cases, post progression sequencing of the FGFR2 gene demonstrated *de novo* point mutations that conferred resistance to BGJ298 (39). While certainly a small study, this publication provides insight into the significant potential of ctDNA analysis to monitor and predict treatment responses by evaluating the accumulation of mutations that confer treatment resistance.

The topic of ctDNA in CCA as a whole remains a vastly underexplored area, yet one with significant clinical potential. The difficulty in obtaining adequate tissue biopsies provides a challenge not only to obtain molecular characterization but also to confirm malignancy. With our recently enhanced understanding of the genomics of this disease and the real, emerging options of targeted therapies for a number of the genomic subtypes of CCA, ctDNA continues to be a tantalizing option for tumor characterization and monitoring, but significant study is necessary going forward to realize this potential.

## Conclusion

Hepatobiliary malignancies are uncommon and devastating malignancies whose incidences are on the rise globally. Though the current literature is quite limited, ctDNA is a promising tool with great potential for application in the detection and management of these malignancies. This review provides a summary of our existing knowledge regarding circulating DNA in the realm of HPB malignancies and seeks to highlight the potential of this tool in these patients. Ongoing and future investigations are encouraged and should seek to prove ctDNA’s capabilities in patients suffering from and those at risk for these devastating diseases.

## Author Contributions

Conceptualization; manuscript writing and editing: KM and SC.

## Conflict of Interest Statement

The authors declare that the research was conducted in the absence of any commercial or financial relationships that could be construed as a potential conflict of interest.
